# Effects of white light‐emitting diode (LED) exposure on retinal pigment epithelium *in vivo*

**DOI:** 10.1111/jcmm.13255

**Published:** 2017-06-29

**Authors:** Imene Jaadane, Gloria Elisa Villalpando Rodriguez, Pierre Boulenguez, Sabine Chahory, Samuel Carré, Michèle Savoldelli, Laurent Jonet, Francine Behar‐Cohen, Christophe Martinsons, Alicia Torriglia

**Affiliations:** ^1^ INSERM U1138 Centre de Recherches des Cordeliers Université Paris Descartes Université Pierre et Marie Curie Paris France; ^2^ ENVA, Ecole Nationale Vétérinaire d'Alfort. Unité d'ophtalmologie Maisons‐Alfort France; ^3^ Division Eclairage et électromagnétisme CSTB, Centre Scientifique et Technique du Bâtiment Saint Martin d'Hères France

**Keywords:** light‐emitting diode, retinal pigment epithelium degeneration, necrosis, oxidative stress, blue light, blood–retinal barrier

## Abstract

Ageing and alteration of the functions of the retinal pigment epithelium (RPE) are at the origin of lost of vision seen in age‐related macular degeneration (AMD). The RPE is known to be vulnerable to high‐energy blue light. The white light‐emitting diodes (LED) commercially available have relatively high content of blue light, a feature that suggest that they could be deleterious for this retinal cell layer. The aim of our study was to investigate the effects of “white LED” exposure on RPE. For this, commercially available white LEDs were used for exposure experiments on Wistar rats. Immunohistochemical stain on RPE flat mount, transmission electron microscopy and Western blot were used to exam the RPE. LED‐induced RPE damage was evaluated by studying oxidative stress, stress response pathways and cell death pathways as well as the integrity of the outer blood–retinal barrier (BRB). We show that white LED light caused structural alterations leading to the disruption of the outer blood–retinal barrier. We observed an increase in oxidized molecules, disturbance of basal autophagy and cell death by necrosis. We conclude that white LEDs induced strong damages in rat RPE characterized by the breakdown of the BRB and the induction of necrotic cell death.

## Introduction

The RPE is a specialized monolayer of hexanocuboidal cells lying between the photoreceptors and the choriocapillaris. RPE cells are interconnected by tight and adherents junctions that isolate photoreceptors from fenestrated capillaries of the choroid, forming a selective barrier called the outer BRB 1. RPE participates in phagocytosis of spent photoreceptors’ outer segments (OS) 2, transepithelial transport of nutrients and waste products 3, visual cycle 2, protection against light and oxidative stress 4, as well as to the production of growth factors. Structural or physiological dysfunction of the RPE leads to retinal diseases, such as AMD 5.

Although it is generally accepted that the primary cause of AMD is the degeneration of the RPE 6, the causes of this degeneration are less understood. Different genetic and environmental factors, like smoking, can lead to the degeneration of the RPE. The influence of light exposure has been widely discussed, but it is now established that light is an important factor in the progression of AMD 7, 8, 9.

During the lifespan, the RPE receives different amounts of natural and artificial light that can induce photochemical lesions. The damages depend on the exposure time, the radiation intensity but also the light's wavelength 10. RPE is very sensitive to blue light that induces oxidative damage and endoplasmic reticulum (ER) stress 11; two lesions also described in the pathogenesis of AMD.

Recently, the use of LED in general lighting has raised concerns regarding the effects of this light source on the RPE. Despite the advantages of these devices, such as low energy consumption and long life, some LEDs potentially produce retinal toxicity due to their intense emission in the blue region of the spectrum 12. We have previously shown that LED light caused retinal injury, including the activation of apoptosis and necrosis and that the blue component of LED emitted light is the major cause of this damage 13, 14.

The exposure limit values (ELV) are the internationally recommended values for the evaluation of the optical radiations toxicity 15, 16, 17. They were calculated on the basis of a factor of 10‐ to 100‐fold lower than the values that can induce observable macroscopically retinal lesions. Concerning the retinal “blue light hazard”, ELV were derived from a basic restriction of 2.2 J/cm^2^ in terms of retinal exposure dose 10. This represents one‐fifth of the calculated toxic dose evaluated at 11 J/cm^2^. Previous studies suggested that these recommendations are not adapted to LED 13 because LED light causes oxidative damage and a loss of photoreceptors without any visible alteration at fundus examination.

This study focuses on the effect of white LED light on the RPE. We investigate, in rats, LED‐induced RPE cell damage using commercially available warm white LED sources, which are widely used in residential lighting. The used doses were intentionally fixed under the damage threshold used for the calculation of the ELV.

## Materials and methods

### Animals

All procedures were performed on 6‐week‐old male Wistar rats (Janvier Laboratories Le Genest‐Saint‐Isle, France) in accordance with the animal use and care committee of the National Veterinary School of Alfort, France, and were approved by its ethical committee.

### Light source

#### LED source

The light device was built and characterized by the lighting and electromagnetism division of the Scientific and Technical Center for Building (CSTB, Saint Martin d'Heres, France): it contains ten white LED lamps (Xanlite Evolution 5 W). The device is fitted with a diffuser improving the uniformity and protecting against the effect of point sources (Fig. S1). The resulting luminance and radiance are, respectively, 2680 cd/m^2^ and 8.33 W/m^2^/sr^1^ 13. The dose of retinal radiant exposure was calculated using dedicated software developed by the CSTB. The presence of the cage was taken into account. The retinal dose was evaluated according to Sliney *et al*. 18 and Van Norren *et al*. 19, taking into account the rat spectral transmittance of the ocular media:

$$H=pt\frac{\pi d^{2}}{4f^{2}}\int_{0}^{\infty }\tau_{\lambda }L_{\lambda }d\lambda \;\left[J/{\text{cm}}^{2}\right].$$

*p* is a posture coefficient, *t* the exposure time, *d* the diameter of the pupil, *f* the focal length, τ_λ_ the spectral transmittance of the rat ocular media, and $L_{\lambda_{i}}$ the spectral radiance of the source. The value for *p* was chosen at 0.5. The values for *d* and *f* were chosen according to Van Norren *et al*. 19 (*d* = 5 mm, *f* = 5.25 mm), in coherence with the values found in Hugues *et al*. 20. Other values for *d* were found in Block 21 (0.4 mm ≤ *d* ≤ 1.2 mm) and Sliney 18 (*d* = 0.5 mm). It is worth noting that in the cited studies, the pupil of the rat was dilated during light exposure. In this study, the pupil is not dilated but we choose 5 mm because we used albino rats and presumed then that their iris did not absorb much light. Following a discussion with Pr van Norren, the value for τ_λ_ was estimated by associating the rat spectral transmittance proposed by the Gorgels *et al*. study 22, with the human cornea transmission published by Van den Berg *et al*. 23.

#### Fluorescent source

Same age and strain rats were exposed to a constant light for 6 hrs and 25 min. at an illumination level of 2000 lx provided by white fluorescent bulbs suspended 20 cm above the transparent polycarbonate cages. This corresponds to a retinal dose of 4.14 J/cm^2^.

### Light exposure

Wistar rats were kept in transparent cages placed under the light source. Without dilated pupils or dark‐adapted, in order to avoid the introduction of procedures inducing photodamage sensitization, the rats were exposed to a constant light for 4.75, 6, 12, 18 and 24 hrs. After exposure to light, the rats were killed using sodium pentobarbital (Ceva, La Ballastiere, France) at lethal dose by intraperitoneal injection. Their eyes were immediately enucleated and dissected for immunofluorescence or biochemical analysis of RPE.

### Inhibition of PKC zeta

The rats were anesthetized with xylazine (Rompon^®^, Bayer Pharma, Puteaux, France) at 10 mg/kg and ketamine (Ketamine Virbac 500 ^®^, Vibrac France, Carros, France) at 40 mg/kg (intramuscular). A local anaesthesia is performed by an instillation of oxybuprocaine (Cebesine ^®^, Laboratoire Chauvin (Bausch and Lomb) Montpellier, France). The injection of PKC zeta inhibitor (myr‐DIYRRGARRWRKL, ref. 539624, Calbiochem) is carried out in the vitreous under a surgical microscope using a 30‐G needle (BD Micro‐Fine, Becton Dickinson S.A., Le Pont de Claix, France). 6 μg of inhibitor was injected in a volume of 10 μl per eye.

### Tissue preparation

After enucleation, the eyes were rinsed with phosphate‐buffered saline (PBS). Some eyes were immediately embedded in optimal cutting temperature (OCT) for preparation of cryosections. In other eyes, the anterior eye portion was removed, and the posterior eyecup was flattened by performing four incisions. The neural retina was separated, revealing the RPE. For immunofluorescence, the whole mount was used. For biochemical analysis, the RPE cells were dissociated and lysed in an extraction reagent for Western blot (WB). Contamination by the neuroretina or choroid was assessed by WB using anti‐rhodopsin and anti‐CD31 antibodies. An additional control was done with the antibody directed against RPE65 protein.

### Protein extraction from light‐exposed retinal pigment epithelium

RPE from two eyes were homogenized in 50 μl of M‐PER extraction reagent (Thermo Scientific, Illkirch, France) with protease and phosphatase inhibitors (protease inhibitor cocktail, Roche, Boulogne‐Billancourt, France) (phosphatase inhibitor cocktail 3, Sigma‐Aldrich, Saint‐Quentin Fallavier, France). The homogenate was then incubated on ice for 15 min. and centrifuged (15,000 g, 4°C) for 15 min. The supernatant's proteins were measured using the bicinchoninic acid (BCA)™ Protein Assay Kit (Thermo Scientific), according to the manufacturer's instructions. Bovine serum albumin (BSA) was used as standard.

### Western blot

Proteins were diluted in Laemmli sample buffer, separated in a 12%, or 10% SDS–PAGE, immobilized on nitrocellulose membrane (Protran^®^, Whatman ^®^, GE Healthcare, Versailles, France) and blotted with specific primary antibody at 1/1000 dilution. The antibodies used are listed in Table 1. The secondary antibodies conjugated to horseradish peroxidise (HRP) (Vector, Eurobio, Les Ulis, France) were used in a 1/5000 dilution. Luminata Forte Western HRP substrate (Millipore, Merck Chimie, Fontenay sous Bois, France) was used to reveal the signal.

**Table 1:** Antibodies used in experiments

Antibody	Source	Application	Reference
Actin‐β (C4)	mouse	WB	Santa Cruz (sc‐47778)
Albumin (N18)	goat	IHC	Santa Cruz (sc‐46291)
CHOP (9C8)	mouse	WB	Thermo scientific (MA1‐250)
Lamin B	goat	WB/IHC	Santa Cruz (sc‐6216)
LAMP2	rabbit	IHC	Sigma‐Aldrich (PRS3627)
LAMP2	rat	WB	Millipore (MABC40)
LC3 (F14)	goat	IHC	Santa Cruz (sc‐16756)
LEI/L‐DNase II	rabbit	WB	Home‐made
NFkB p65 (H286)	rabbit	WB	Santa Cruz (sc‐7151)
P62/SQSTM1	mouse	WB/IHC	Abcam (ab56416)
Phospho‐NFkB p65 (S311)	rabbit	WB/IHC	Abcam (ab‐51059)
Phospho‐PKC zeta (Thr 410)	rabbit	WB	Santa Cruz (sc‐12894R)
PKC zeta (C20)	rabbit	WB/IHC	Santa Cruz (sc‐216)
RIP (receptor‐interacting protein)	mouse	WB/IHC	BD (610459)
XBP1 (M186)	rabbit	WB	Santa Cruz (sc‐7160)
RPE65	mouse	WB	Thermo scientific (MA1‐16578)

WB, Western blot; IHC, immunohistochemistry.

### Immunofluorescence

Cryosections of the eyes or flat mounts of the RPE were fixed in 4% paraformaldehyde for 15 min., permeabilized with 0.3% Triton X‐100 for 20 min. and blocked by 1‐hr incubation with 1% non‐fat milk in PBS. Specific primary antibodies were diluted in 0.1% non‐fat milk in PBS (1/100) and incubated for 1 hr. The antibodies used are shown on Table 1. Specific secondary antibodies (1/500 dilution) were incubated 1 hr. Finally, samples were incubated 20 min. with Fluor 594 phalloidin (Santa Cruz, SC‐363795 Dallas, Texas, USA) 1/1000 and for 5 min. with 4,6 di‐aminidino‐2‐phenyl indoledichloride DAPI 1/5000 (Sigma‐Aldrich). Immunoreactivity was visualized using an Olympus fluorescence microscope or a Zeiss LSM 710 confocal microscope (Carl Zeiss Microscopy GmbH, Munich, Germany). It is important to note that the lesions seen in the RPE were not uniformly distributed. We concentrate on the superior pole, at 100 μm from the optic nerve to evaluate the lesions.

### Transmission electron microscopy analysis (TEM)

The eyes were fixed in 4% glutaraldehyde cacodylate buffer (0.1 M, pH 7.4) for 5 hrs, in 1% osmium tetroxide in cacodylate buffer (0.2 M, pH 7.4) and progressively dehydrated in graduated ethanol solution (50, 70, 95 and 100%) then in propylene oxide. Each area of interest was separated in four samples, included in epoxy resin and oriented. Semi‐thin sections (800 nm) were obtained with an ultra microtome (LEICA Ultracut UCT Austria) and stained with toluidine blue. Ultra‐thin sections (80 nm) were contrasted by uranyl acetate and lead citrate and analysed with a transmission electron microscope (Philips CM10, the Netherlands) with a GATAN ES100W camera (Pleasanton, CA, USA).

### Measurement of the cell size

Cell size vas evaluated on phalloidin‐labelled flat mounts using the Fiji software for image analysis. A minimum of 100 cells were measured in the dorsal area of the fundus 500 μm away from the optic nerve. Eight eyes were examined for each group. Significance was evaluated using the t‐test between NE eyes and eyes exposed to LED. This was done after normal distribution verification using the test of Kolmogorov–Smirnov, Shapiro–Wilk and D'Agostino‐Pearson.

## Results

### Disruption of the BRB induced by LED

**Figure 1:**
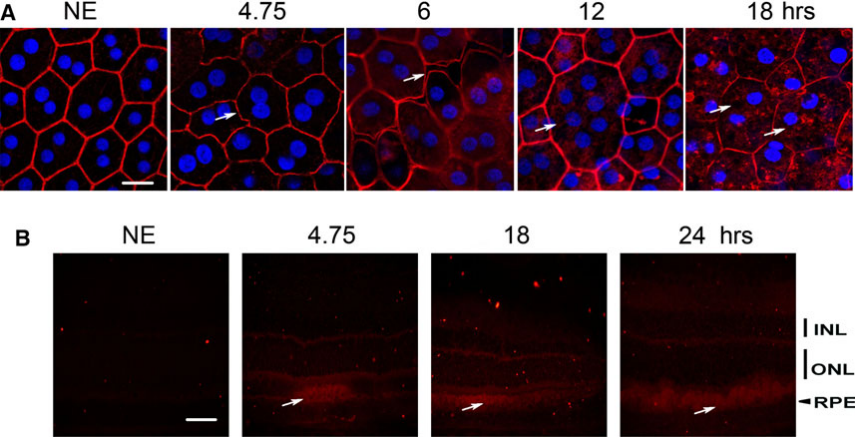
Structural alterations induced by LED exposure. Male Wistar rats aged 7 weeks (*n* = 4) were exposed to white LED for 4.75, 6, 12, 18, 24 hours receiving the retinal doses of light of 4.14, 5.23, 10.5, 15.7 and 20.9 J/cm^2^, respectively. NE: Non‐exposed rats. (**A**) At the end of the exposure period, the RPE was flat‐mounted and stained with phalloidin, which binds to polymerized actin (red) and with DAPI (blue) that labels the nuclei. The white arrows point alterations of the actin cytoskeleton. The RPE was analysed by confocal microscopy. The pictures were taken on the upper retina 100 μm away from the optic nerve. Scale bar represents 10 μm. (**B**) After LED exposure, the eyes were included in optimal cutting temperature medium (Tissue Tek), cryosectioned and immunostained with anti‐albumin (red). The white arrows point infiltration of albumin in the neuroretina. ONL: outer nuclear layer; INL: inner nuclear layer; RPE: retinal pigment epithelium. Scale bar represents 20 μm.

The effect of white LED light exposure on RPE was first explored by staining the actin cytoskeleton using phalloidin which binds to polymerized actin. We observed a disorganization of the cytoskeleton after 4.75 hrs of LED exposure (4.14 J/cm^2^) (Fig. 1A). After 6 hrs of exposure (5.23 J/cm^2^), there was a disruption of tight junctions with changes in cell morphology and appearance of stress fibres. After 12 hrs of exposure (10.5 J/cm^2^), syncytia appeared. For longer exposures (18 hrs, 15.7 J/cm^2^), actin became sparse and present cytoplasmic aggregates (Fig. 1A). Some cells almost completely lost their actin cytoskeleton and presented many vacuoles (Fig. 1A, 18 hrs, white arrows). These alterations and mostly the opening between cells suggested a loss of the BRB. This was confirmed by the leakage in the interphotoreceptor space of serum albumin on retina cryosections (Fig. 1B, white arrows) that increased with the time of exposure.

### Damages and oxidative stress induced by LED

**Figure 2:**
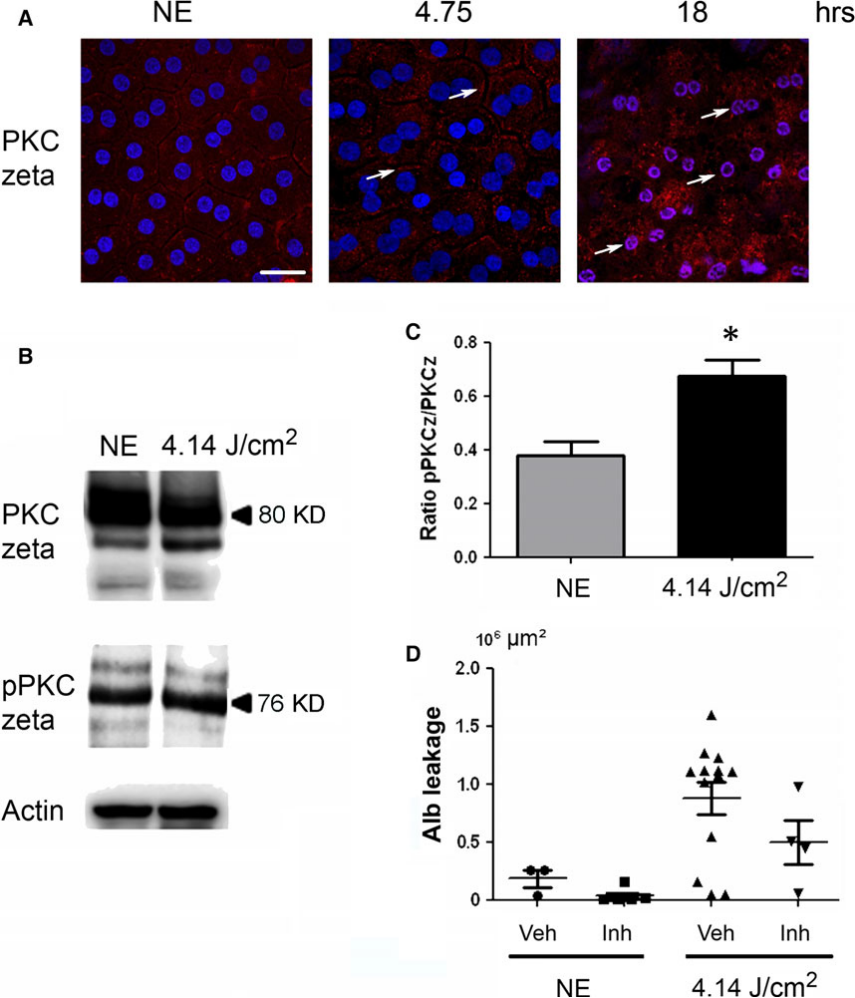
The activation of PKC zeta after LED exposure. Male Wistar rats aged 7 weeks (*n* = 4) were exposed to white LED for 4.75 hrs and 18 hrs. NE: Non‐exposed rats. (**A**) At the end of the exposure period, the RPE was flat‐mounted and immunostained with anti‐PKC zeta and DAPI then examined by confocal microscopy. The white arrows point PKC zeta located at the plasma membrane (4.75 hrs) and at nuclear localization (18 hrs). The pictures were taken on the upper retina 100 μm away from the optic nerve. Scale bar represents 20 μm. (**B**) After 4.75 hrs (4.14 J/cm^2^) of light exposure, the eyes were enucleated, and the RPE dissected, extracted with M‐PER buffer and loaded on the top of a 10% SDS–PAGE, transferred onto a nitrocellulose membrane and probed for anti‐PKC zeta (upper panel), anti‐phospho‐PKC zeta (middle panel) and anti‐actin antibody as a charge control (lower panel). (**C**) The histogram shows a quantification of PKC zeta phosphorylation expressed as a ratio to PKC zeta normalized to actin. Values are significantly different *=*P* < 0.05. (**D**) The inhibition of PKC zeta induced a decrease in the leakage of albumin as shown by the decrease in the number of leakages (each point) and the decrease of their size. This is the result of a representative experiment; Vhe: vehicle, Inh: PKC zeta inhibitor.

**Figure 3:**
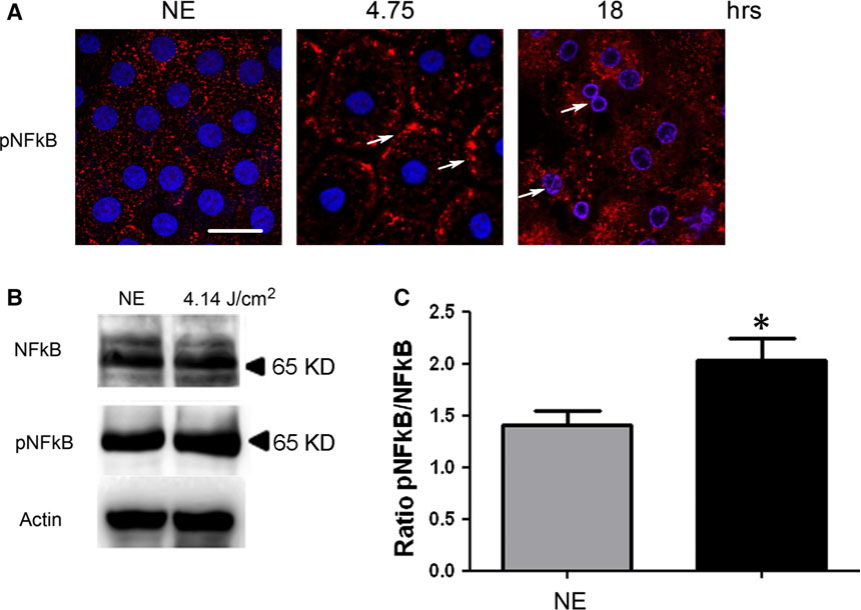
NFkB activation after LED exposure. Male Wistar rats aged 7 weeks (*n* = 4) were exposed to white LED for 4.75 hrs and 18 hrs. NE: Non‐exposed rats. (**A**) At the end of the exposure period, the RPE was flat‐mounted and immunostained with anti‐phospho‐NFkB (ser 311). The RPE was further analysed by double staining with DAPI by confocal microscopy. Scale bar represents 20 μm. The white arrows point pNFkB at the plasma membrane (4.75 hrs) and nuclear localization (18 hrs). (**B**) After 4.75 hrs (4.14 J/cm^2^) of light exposure, the eyes were enucleated, the RPE dissected, extracted with M‐PER buffer and loaded on the top of a 10% SDS–PAGE, transferred onto a nitrocellulose membrane and probed for anti‐NFkB (upper panel), anti‐phospho‐NFkB (ser 311) (middle panel) and anti‐actin antibody as a charge control (lower panel). (**C**) The histogram shows a quantification of NFkB phosphorylation expressed as a ratio to NFkB normalized to actin. Values are significantly different *=*P* < 0.1.

As the oxidation of the different components (proteins, lipids, etc.) was difficult to label in RPE (not shown), we used indirect signs of oxidative stress by studying its cellular responses. PKC zeta, an atypical protein kinase 24, was activated after 4.75 hrs of LED exposure as indicated by its plasma membrane translocation 25, 26, 27 (Fig. 2A, arrows). This location is progressively lost and after 18 hrs of exposure, and PKC zeta is translocated to the nucleus (Fig. 2A, white arrows, co‐labelling with DAPI (blue)/PKC zeta (red)). This nuclear translocation follows PKC zeta cleavage 28 and has already been described as a sign of cell death. This was confirmed by WB against phospho‐PKC zeta (Fig. 2B and C) and against its main target, the transcription factor NFkB 25, 29 (Fig. 3). After 4.75 hrs of LED light exposure, we observed a plasma membrane accumulation of phospho‐NFkB (Fig. 3A, arrows). After 18 hrs of exposure, NFkB was nuclear translocated (Fig. 3A, co‐staining blue/red). We verified this activation by WB (Fig. 3B and C).

Interestingly, the activation of PKC zeta in the early‐phases of diabetes has been shown to be involved in the opening of the BRB. Actually, its inhibition partially restored the barrier function of the RPE in a rat model of diabetes mellitus 29. To investigate whether this was also the case here, we inhibited PKC zeta as before 27. The leakage of the BRB was quantified by measuring the surface of the rat serum albumin seen at the photoreceptors segments layer (see Fig. 2D). The results show that inhibition of PKC zeta (confirmed by the decreased phosphorylation of NF‐kB on serine 311) decreased albumin leakage.

**Figure 4:**
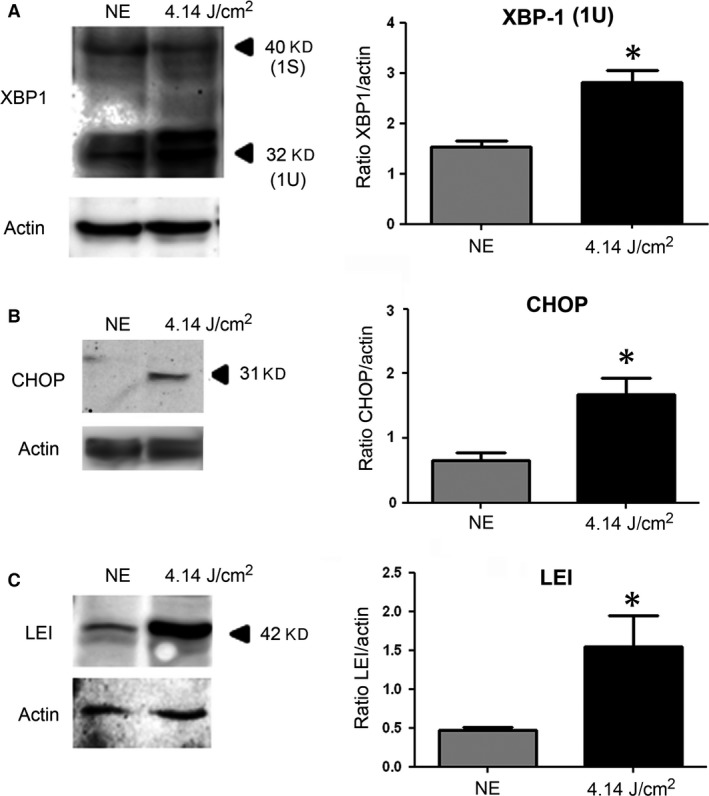
Western blot analysis of mechanisms of stress response after LED exposure. Male Wistar rats aged 7 weeks (*n* = 4) were exposed to white LEDs for 4.75 hrs. NE: Non‐exposed animals. At the end of the exposure period, the eyes were analysed regarding different effectors of stress response. After light exposure, the eyes were enucleated, the RPE dissected, extracted with M‐PER buffer and loaded on the top of a 12% SDS–PAGE, transferred onto a nitrocellulose membrane and probed for different antibodies. (**A**) WB using anti‐XBP1 antibody. The histogram to the right shows a quantification of XBP‐1U protein expressed as a ratio to actin. Values are significantly different *P* < 0.05. (**B**) Same experiment that in panel A using anti‐CHOP antibody. The histogram to the right shows a quantification of CHOP protein expressed as a ration to actin *=(*P* < 0.05). (**C**) Same experiment using anti‐LEI‐derived DNase II (LEI/L‐DNase II) with its quantification by histogram (*P* < 0.05). Actin was used as a charge control in the different experiments.

To evaluate the endoplasmic reticulum stress response (ER stress), two members of this pathway were investigated through the activation of XBP1 (X‐box binding protein) and CHOP (CCAAT/‐enhancer‐binding protein homologous protein). We found a statistically significant increase of XBP‐1 (Fig. 4A) and CHOP (Fig. 4B), indicating an activation of the ER stress response.

Oxidative stress leads also to an activation of proteases counterbalanced by the synthesis of anti‐proteases like LEI (leucocyte elastase inhibitor), which is increased after LED exposure (Fig. 4C).

### Disturbance of autophagy induced by LED light exposure

Autophagy is a cellular process, very important in cell housekeeping involved in the removal of damaged organelles and protein aggregates 30. We evaluated its status by investigating three molecules involved in this process: p62, LC3 and LAMP2.

**Figure 5:**
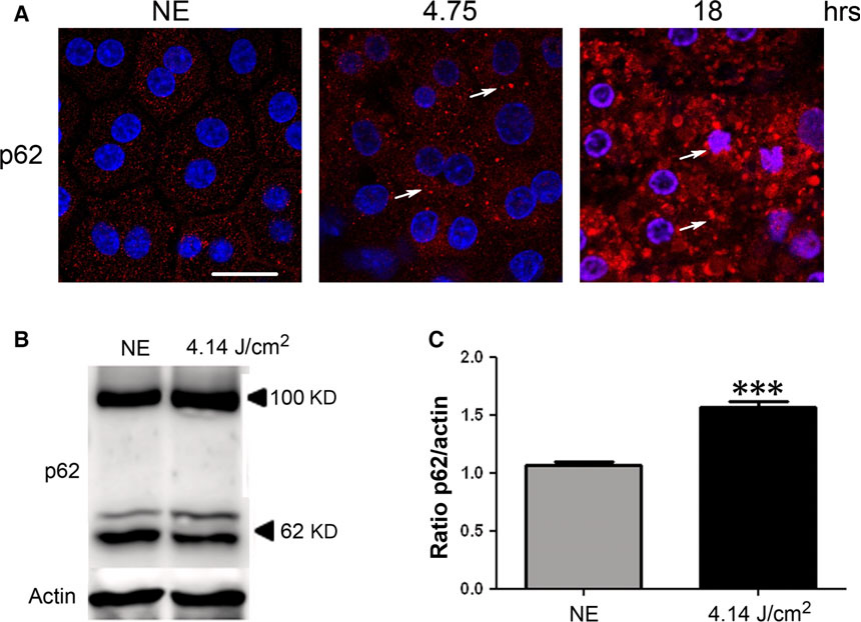
Oxidative stress and disturbance of autophagy flow after LED exposure. Male Wistar rats aged 7 weeks (*n* = 4) were exposed to white LED for 4.75 hrs and 18 hrs. NE: Non‐exposed rats. (**A**) At the end of the exposure period, the RPE was flat‐mounted and immunostained with anti‐p62. The RPE was further analysed by double staining with DAPI by confocal microscopy. The pictures were taken on the upper retina 100 μm away from the optic nerve. Scale bar represents 20 μm. The white arrows point p62 cytoplasmic clusters (4.75 hrs) and nuclear localization (18 hrs). (**B**) After 4.75 hrs (4.14 J/cm^2^) of light exposure the eyes were enucleated, the RPE dissected, extracted with M‐PER buffer and loaded on the top of a 10% SDS–PAGE, transferred onto a nitrocellulose membrane and probed for anti‐p62 (upper panel) and anti‐actin antibody as a charge control (lower panel). (**C**) The histogram shows a quantification of p62 expressed as a ratio to actin. Values are significantly different ***=*P* < 0.005.

**Figure 6:**
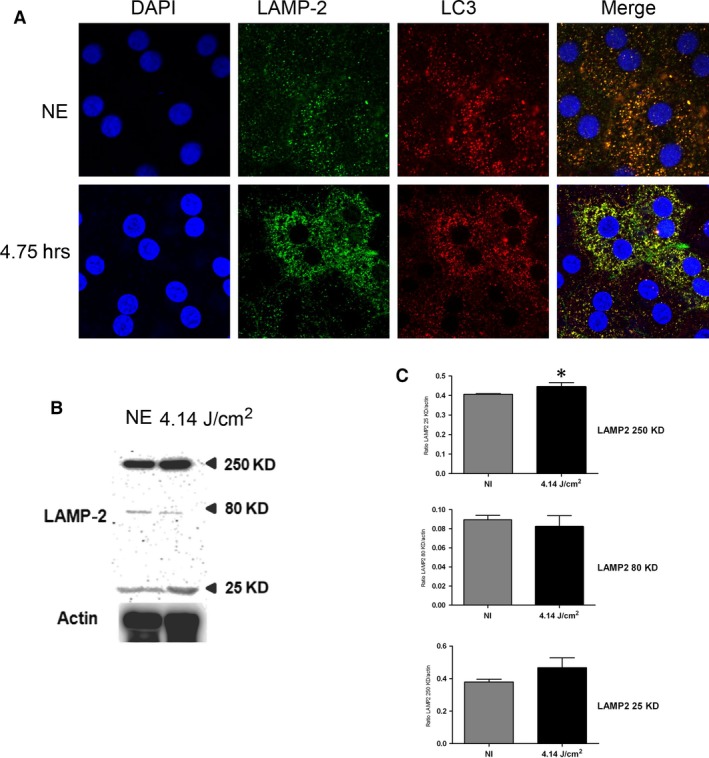
Physiological function of autophagy after LED exposure**.** Male Wistar rats aged 7 weeks (*n* = 4) were exposed to white LED for 4.75 hrs. NE: Non‐exposed rats. (**A**) At the end of the exposure period, the RPE was flat‐mounted and immunostained with two combinations of antibodies: anti‐LC3 in red and with anti‐LAMP2 in green. The RPE was further analysed by triple staining with DAPI (blue) by confocal microscopy. The pictures were taken on the upper retina 100 μm away from the optic nerve. Scale bar represents 20 μm. (**B**) After LED exposure, the eyes were enucleated, the RPE dissected, extracted with M‐PER buffer and loaded on the top of a 10% SDS–PAGE, transferred onto a nitrocellulose membrane and probed for anti‐LAMP2. Actin was used as a charge control. (**C**) The histogram shows a quantification of p62 expressed as a ratio to actin * =*P* < 0.05).

P62 (sequestosome 1) is continuously degraded by autophagy, and its synthesis is increased during oxidative stress 31, 32. After 4.75 hrs of LED exposure, p62 accumulates in clusters (Fig. 5A, arrows, and Fig. 5B and C). After 18 hrs, a nuclear localization of the protein suggested an alteration of nucleo‐cytoplasmic traffic 33 and a slowdown of autophagy. To confirm this, we investigated LAMP2 (lysosome‐associated membrane protein 2) that plays an essential role in the fusion between autophagosome and lysosome 34 and LC3 (light chain 3), a protein of the autophagosome membrane. In non‐exposed RPE, most of LAMP2 co‐localize with LC3 indicating the presence of many autophagolysosomes (Fig. 6A). After LED exposure (4.75 hrs), this staining increased, suggesting an enlargement of the size of lysosomes and a decrease of autophagy. Moreover, the WB of LAMP2 showed an increase in the glycosylated form (250 kD) (Fig. 6B and C) which may suggest a disturbance in the LAMP2 maturation 35 but also an inefficient fusion between autophagosome and lysosome. In addition, a cleaved form of LAMP2 appeared in exposed rats suggesting some extent of lysosomal permeabilization (LMP) 36.

### RPE degeneration induced by white LED exposure

**Figure 7:**
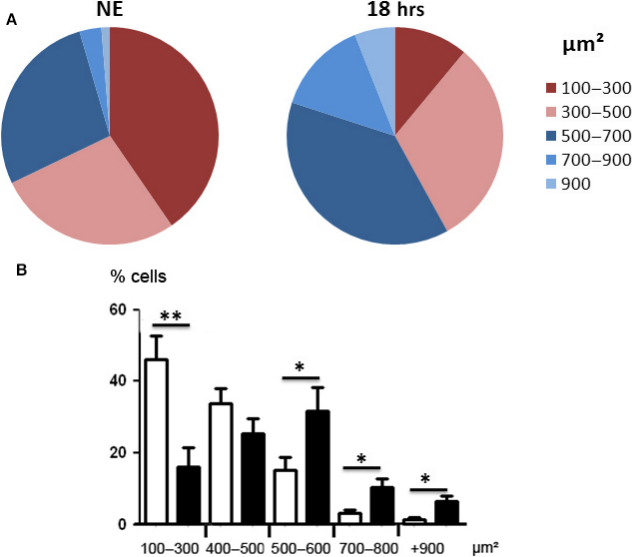
Distribution of cell sizes of the RPE after LED exposure. Male Wistar rats aged 7 weeks (*n* = 4) were exposed to white LED for 18 hrs. NE: Non‐exposed rats. At the end of the exposure period, the RPE was flat‐mounted and stained with phalloidin, which binds to polymerized actin. An analysis of the number of cells depending on their cell surface was done by confocal microscopy using the Fiji software, eight eyes were analysed, and at least 100 cells/eye were evaluated. Sector diagram (**A**) shows the distribution of cells according to their size. The statistical analysis is shown in the lower histogram (**B**). (*P < 0.05; **P < 0.01). White bars represents control conditions (rats non‐exposed to LED), and black bars represent rats exposed to LED.

**Figure 8:**
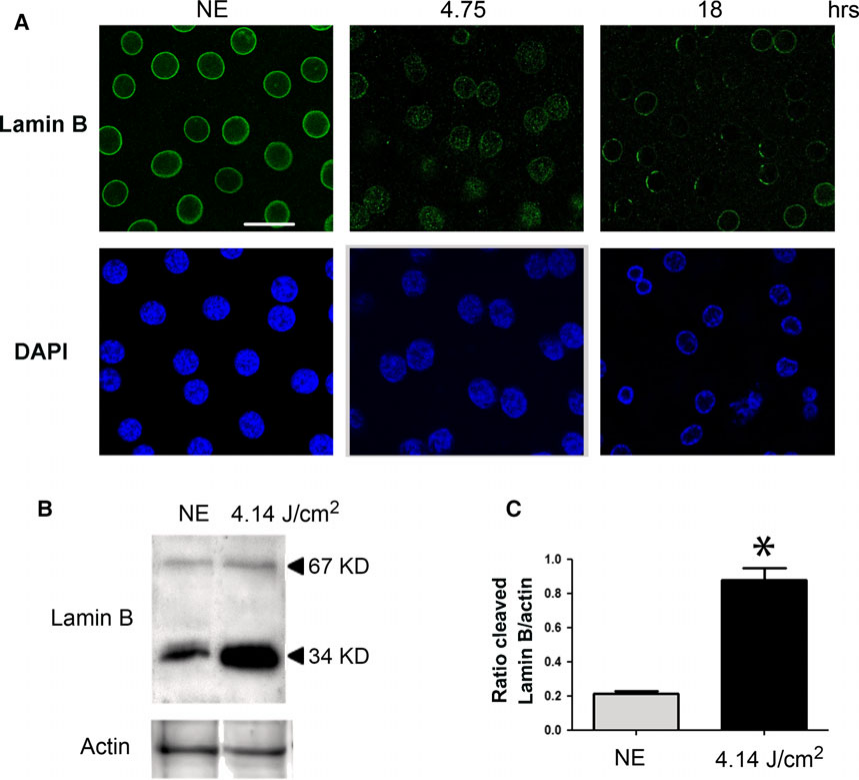
RPE degeneration after LED exposure. Male Wistar rats aged 7 weeks (*n* = 4) were exposed to white LED for 4.75 hrs and 18 hrs. NE: Non‐exposed rats. (**A**) At the end of the exposure period, the RPE was flat‐mounted and immunostained with anti‐Lamin B in green and with DAPI by confocal microscopy. The pictures were taken on the upper retina 100 μm away from the optic nerve. Scale bar represents 20 μm. (**B**) After LED exposure, the eyes were enucleated; the RPE dissected, extracted with M‐PER buffer and loaded on the top of a 12% SDS–PAGE, transferred onto a nitrocellulose membrane and probed for anti‐Lamin B. Actin was used as a charge control. (**C**) The histogram shows a quantification of Lamin B expressed as a ratio to actin. Values are significantly different *=*P* < 0.05.

Apoptotic cells were not observed. This could be related to the capacity of RPE to expand in order to avoid the interruption of the outer BRB, as verified by calculating the surface of the cells. Figure 7 shows a shift of surfaces of the RPE towards larger cells. Moreover, apoptosis might not be the preferred form of cell death in RPE and was not observed (see TUNEL staining of retinas from exposed rats, Fig. S2, where the damaged neural retina (revealed by the presence of TUNEL‐positive cells in the ONL) faced TUNEL‐negative RPE)) 37. A chromatin condensation was observed at the periphery of the nuclei after 4.75 hrs of LED exposure (Fig. 8A). After 18 hrs, chromatin was more condensed and nucleus size was reduced. Immunostaining of Lamin B (Fig. 8A) becomes discontinuous and after 18 hrs of LED exposure almost disappeared. The degradation of Lamin B was confirmed by WB (Fig. 8B and C). Taken together, these elements suggested the activation of necrosis that was further investigated.

**Figure 9:**
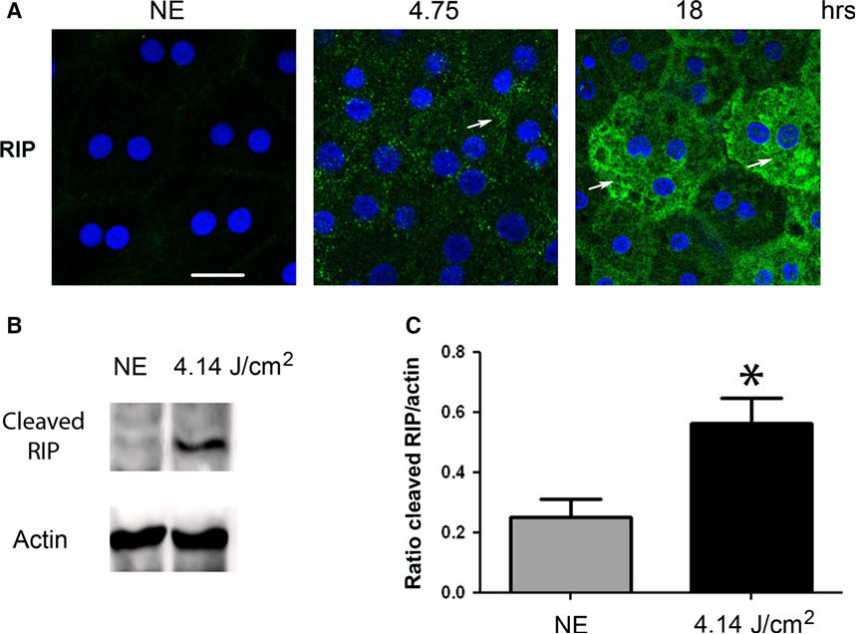
The mechanisms of cell death in RPE after white LED exposure. Male Wistar rats aged 7 weeks (*n* = 4) were exposed to white LED for 4.75 hrs and 18 hrs. NE: Non‐exposed rats. (**A**) At the end of the exposure period, the RPE was flat‐mounted and immunostained with anti‐RIP in green and with DAPI then analysed by confocal microscopy. The pictures were taken on the upper retina 100 μm away from the optic nerve. Scale bar represents 20 μm. (**B**) After LED exposure, the eyes were enucleated, the RPE dissected, extracted with M‐PER buffer and loaded on the top of a 10% SDS–PAGE, transferred onto a nitrocellulose membrane and probed for anti‐RIP. Actin was used as a charge control. (**C**) The histogram shows a quantification of cleaved RIP expressed as a ratio to actin. Values are significantly different *=*P* < 0.05.

**Figure 10:**
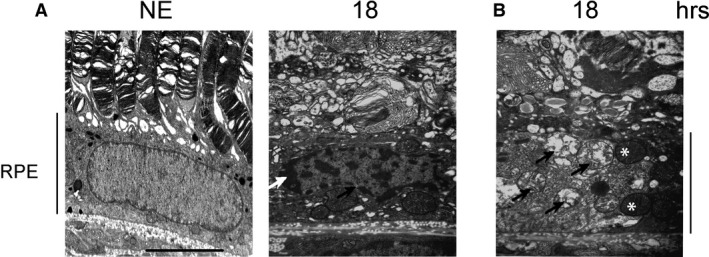
Structural modifications of the RPE examined by transmittance electron microscopy (TEM). Male Wistar rats aged 7 weeks (*n* = 4) were exposed to white LED for 18 hrs (15.7 J/cm^2^). At the end of the exposure period, the eyes were analysed by TEM. NE: Non‐exposed animals. (**A**) After light exposure, the RPE presents peripheral condensation of chromatin (white arrow) and dilated nuclear pores (black arrow). (**B**) LED exposure leads to an alteration of the mitochondria. The black arrows point highly damaged mitochondria. The white stars indicate condensed mitochondria. The pictures were taken on the upper retina 100 μm away from the optic nerve. Scale bar represents 5 μm. RPE: retinal pigment epithelium.

To evaluate the activation of programmed necrosis, we studied RIP (receptor‐interacting protein kinase) 38. After 4.75 hrs of LED exposure, the staining of RIP revealed an accumulation in some cytoplasmic clusters (Fig. 9A). After 18 hrs, we observed an intense labelling in scattered groups of cells, a distribution commonly featured in necrosis (Fig. 9A). The cleavage of RIP observed by WB after LED light exposure confirms activation of necrosis (Fig. 9B and c). These results were enforced by transmission electron microscopy (TEM) that confirmed the condensation of chromatin to the periphery of nuclei revealed the presence of dilated nuclear pores (Fig. 10A) and damage of mitochondria (Fig. 10B).

### The specificity of light coming from LEDs in the development of the RPE damage

To evaluate whether the damages to the RPE described before were produced specifically by the light coming from LED, we submitted rats to fluorescent light at the same retinal dose than used for LED exposure, for example 4.14 J/cm^2^ (Fig. S3). In this case, we did not found any alteration of the actin cytoskeleton, no increase of RIP and, importantly, the BRB kept its barrier function. Actually, the albumin labelling of the slides only showed an increase of the labelling of RPE cells in very scattered areas. Figure S3B shows a “worst case” of these experiments.

## Discussion

The exposure limit values (ELV) fix a restriction of 2.2 J/cm^2^ of retinal exposure dose in 10,000 sec. 10. This represents one‐fifth of the calculated toxic dose evaluated at 11 J/cm^2^. Previous studies suggested that these recommendations are not adapted to LED 13 because LED light causes oxidative damage and a loss of photoreceptors without any visible alteration at fundus examination. The results presented here are in agreement with this because we show damages at doses lower than 11 J/cm^2^. Moreover, the dose of 4.14 J/cm^2^ used in this study was not delivered in 10,000 sec. but in 64,800 sec. If only the blue part of the spectrum is taken into account, the retinal dose of blue light represents 14% of the total spectrum, that is 0.58 J/cm^2^. Far below, the 2.2 J/cm^2^ presumed to be completely safe. RPE has an important role in the clearing of oxidized photoreceptors outer segments, and the light damage at its level could be responsible for the degeneration of the neural retina, a feature seen in AMD.

Previous studies have described two types of damages induced by light: the first involves rhodopsin and affects photoreceptors 19, 39, 40. The second concerns the RPE which is selectively vulnerable to high‐energy blue light 41, 42. RPE sensitivity to LED lighting was previously explored by Chamorro *et al*. which showed that exposure to LED induces ROS production and cell death in an *in vitro* model of cultured HRPE cells (human retinal pigment epithelial) 43. In our *in vivo* study, we show that after LED exposure, a disorganization of the actin cytoskeleton and a disruption of tight junctions with an infiltration of albumin in the outer retina, indicating a permeabilization of the outer BRB, a feature seen in common retinopathies (diabetic retinopathy, for instance) 29. Moreover, we found early formations of polynucleated cells and an increase of the cells size both features related to ageing of the RPE cells 44, 45.

Concerning cell survival our experiments showed that RPE degeneration induced by LED exposure seems to occur in two phases. An early‐phase (retinal dose: 4.14 J/cm^2^) characterized by signs of oxidative stress, followed by an advanced phase (15.7 J/cm^2^), where cytoskeletal lesions are more conspicuous and necrosis extends to several cells.

In our *in vivo* model, we also find signs of oxidative stress after LED exposure: increasing XBP‐1U leads to up‐regulation of the UPR genes and contributes to the recovery from ER stress, while CHOP induces the expression of pro‐apoptotic factors 46. These results are in agreement with Nakanishi's 47, as well as Zhao's studies on ARPE 19 cells 11.

In the early‐phase, we also saw the induction of the LEI expression, a protein of the serpin superfamily 48. LEI, also known as serpin B1, inhibits elastases, AP24 and cathepsin D 49. This may be part of the oxidative stress response as shown previously 50.

The stress generated by LED light exposure induces also survival‐promoting signals. We show the activation of the PKC zeta/NFkB axis involved in different types of retinal damage 29 13 27. Following stress, activated PKC zeta (phosphorylated at threonine 410) triggers the phosphorylation cascade that leads to the activation of NFkB (by phosphorylation on serine 311) 28, 30, 51. All these forms were shown as activated in our study. In addition, the plasma membrane location of PKC zeta suggests that part of this effort is related to the stability of tight junctions 52. In advanced stages of damage, this kinase shows a nuclear immunostaining corresponding to its cleaved form 53. This biphasic response of PKC zeta has already been described as protective when located at the membrane and promoting cell death when cleaved and nuclearized 54. Concerning p62 (sequestosome 1), it presents also a biphasic behaviour. Shorter times of exposure induced an accumulation of p62 in the cytoplasm. This change, together with the modifications of LC3 and LAMP2, suggests a blocking of autophagy. It is important to note that insufficient digestion of discs due to reduced autophagy or lysosomal impairment in the RPE is reported to lead to accumulation of damaged organelles and toxic proteins including lipofuscin, as well as extracellular Drusen deposits, all of which have been associated with the pathogenesis of AMD 55, 56. After longer exposures, a nuclear localization of p62 is observed. This was described as a disruption of the nuclear‐cytoplasmic shuttling of p62 33 and is a sign of cellular suffering.

Taken together, these results indicate that the stress induced by LED exposure triggers the activation of several stress responses in the RPE cells that are overwhelmed if the exposure is maintained. A similar response was seen in cultured cells exposed to blue light in the Roehlecke *et al*. study 57.

After longer exposures, LED light causes RPE degeneration. We investigated the presence of apoptosis. Caspase‐dependent apoptosis and caspase‐independent apoptosis were investigated. This was done using an anti‐active‐caspase 3 antibody, an anti‐AIF 58 and an anti‐L‐DNase II 59 for caspase‐independent apoptosis. Contrary to what happens in LIRD, 60, 61 none of these effectors were activated in RPE exposed to LED suggesting the involvement of another type of cell death. Actually, the morphology of nuclei, the modification of Lamin B labelling and the RIP staining in scattered groups of cells and its cleavage indicate the activation of necrosis. To note, this type of cell death was also shown in photoreceptors of LED‐exposed animals 13. This necrosis will produce an inflammatory reaction. Although we did not look at this aspect, our results are in accord with Brandstetter's team that showed an activation of the inflammasome and a secretion of interleukin‐1β in ARPE19 cells exposed to blue LED light 62. Moreover, Narimatsu *et al*. showed an increase in inflammatory cytokine expression as well as macrophage recruitment in the RPE‐choroid *in vivo* of mice exposed to blue light 63.

**Figure 11:**
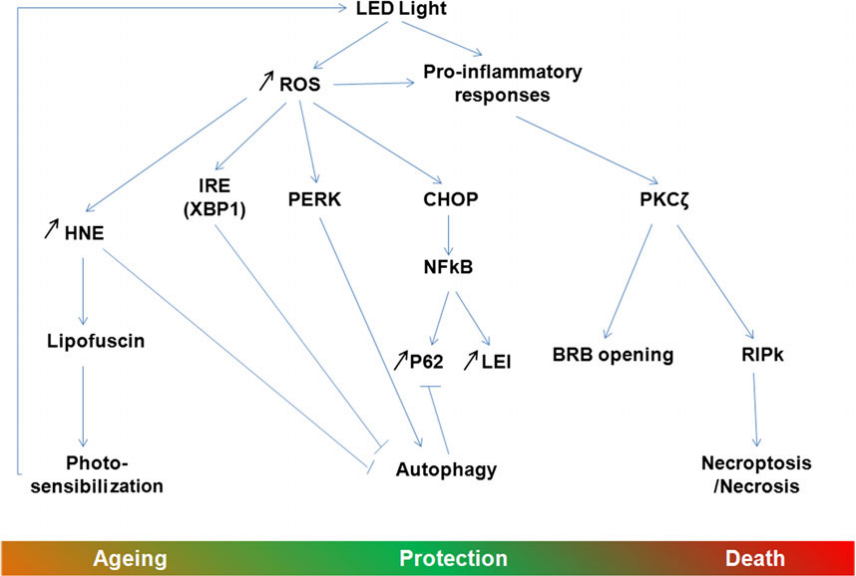
Scheme of the possible sequence of the molecular mechanisms activated in the RPE by LED exposure. ROS: Reactive oxygen species, IRE: inositol requiring enzyme, XBP‐1: X‐box binding protein, PERK: protein endoplasmic reticulum kinase, CHOP: CCAAT enhancer‐binding protein homologous protein. NF‐kB: Nuclear factor kappa B, p62: sequestosome, LEI: leucocyte elastase inhibitor, PKC zeta: protein kinase C zeta, RIPk: receptor‐interacting protein kinase, BRB: blood–retinal barrier, HNE: hydroxynonenal.

Altogether, the obtained results can suggest the following sequences of events (Fig. 11): the oxidative stress produced by the high‐energy light coming from LED induced the production of ROS 64 and a pro‐inflammatory cytokine response 63. The oxidative stress, on the one hand, induces protein modification and misfolding, leading to unfolded protein response (UPR) and ER stress activation 65. The ER stress may induce NF‐kB activation and mitochondrial permeabilization. Moreover, this pathway also modulates autophagy by increasing the synthesis of proteins involved in this process (essentially thought the PERK‐CHOP and ATF6 pathways) or inhibiting the process by the XBP‐1 pathway 66.

On the other hand, pro‐inflammatory cytokines activate the atypical PKC zeta, a feature already seen in retina under light exposure conditions and diabetes 27, 29 and increases the expression of RIP kinases 63, 67. The activation of PKC zeta leads to the opening of the outer blood–retinal barrier, as also seen in diabetes 29. Actually, as in diabetes, the use of a PKC inhibitor restores the outer BRB. PKC zeta, in turn, activates NF‐kB (by phosphorylation on ser 311, as shown here), which will have the actions described above but that will also increase the expression of anti‐apoptotic molecules like many members of the Bcl2 family 68 and of LEI 49. In its native form, LEI can protect the cells against lysosomal permeabilization by inhibiting some lysosomal proteases 49. NF‐kB can also increase the synthesis of p62. This protein participates in many processes and acts as a cargo to drive damaged proteins into the autophagosome 69.

All these cell‐protective mechanisms activated seem to be over helmed as suggested by the increased leaking of the BRB and the beginning of the PKC zeta nuclearization after 6 hrs of LED exposure. Cell fate finally proceeds to necrotic cell death favoured by the probable blockage of autophagy (suggested by the accumulation of p62 and of damaged mitochondria) and by the increase of RIP.

We have previously shown 13 that LED exposure greatly increases lipid peroxidation and HNE production at the photoreceptors segments level. These oxidized lipids are known to increase lipofuscin production in RPE leading to photo‐sensibilization 45, inhibition of lysosomal enzymes 70 leading to an inhibition of autophagy and to an ageing of the RPE cells, all features found in AMD 71.

The results presented here were obtained in rats. Their eyes are different from humans because they are nocturnal animals and they do not possess a macula. The first difference is very important in considering neural retinal but less when considering RPE because their anatomy and physiology are closer. The rat retina does not have a macula, and this region of the human retina devoted to sharp vision and protected by macular pigments. However, during ageing, these pigments are reduced and photosensitive pigments like lipofuscin accumulate increasing the photosensitivity of RPE. So that, although the results presented herein were obtained in an experimental condition and cannot be directly transposed to humans, they raise two important questions: How will the RPE cells manage to control the oxidative stress produced over years of exposure to this type of light sources? And how will repeated exposure affect specific functions such as autophagy and phagocytosis of photoreceptors outer segments?

## Conclusion

In this article, we analysed the effects of white LEDs on the retinal pigment epithelium. We found important structural alterations and damages leading to the disruption of the outer BRB after LED light exposure. At the molecular level, analyses revealed an increase of oxidative stress followed by cell death by necrosis, a rare event in this type of tissue.

The observed injuries of the RPE reinforce our concerns regarding the general and unrestricted use of LEDs in lighting products and the necessity of revising the product safety standards for this type of lighting, with the aim of to protecting our vision during our whole life‐time.

## Author Contributions

IJ and GEVR performed most of the experiments; PB, SC, CM designed, measured and mounted the lighting devices; LJ and MS gave technical support; SC and FBC gave scientific advice; and IJ and AT designed the experiments, analysed the data and wrote the article.

## Conflict of Interest

The authors disclaim that there is not conflict of interest.

## Supporting information


**Figure S1** Light exposure device.

**Figure S2** TUNEL labelling showed no apoptosis in RPE cells.

**Figure S3** RPE from rats exposed to 4.14 J/cm^2^ of fluorescent tubes.
