# Supplementary figures and images for: Effects of white light‐emitting diode (LED) exposure on retinal pigment epithelium in vivo
Source: J Cell Mol Med. 2017 Jun 29;21(12):3453–66. doi: 10.1111/jcmm.13255 (PMC5706508; doi:10.1111/jcmm.13255)

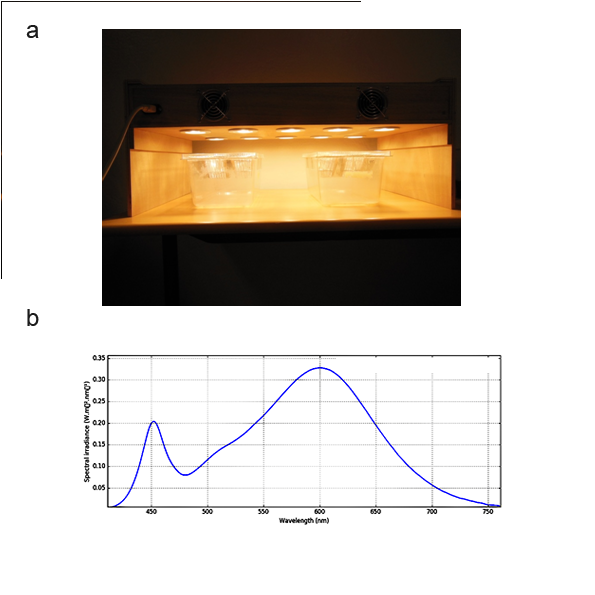

Supplement: Supplementary file 1 — Figure S1 Light exposure device. [file JCMM-21-3453-s001.tif]

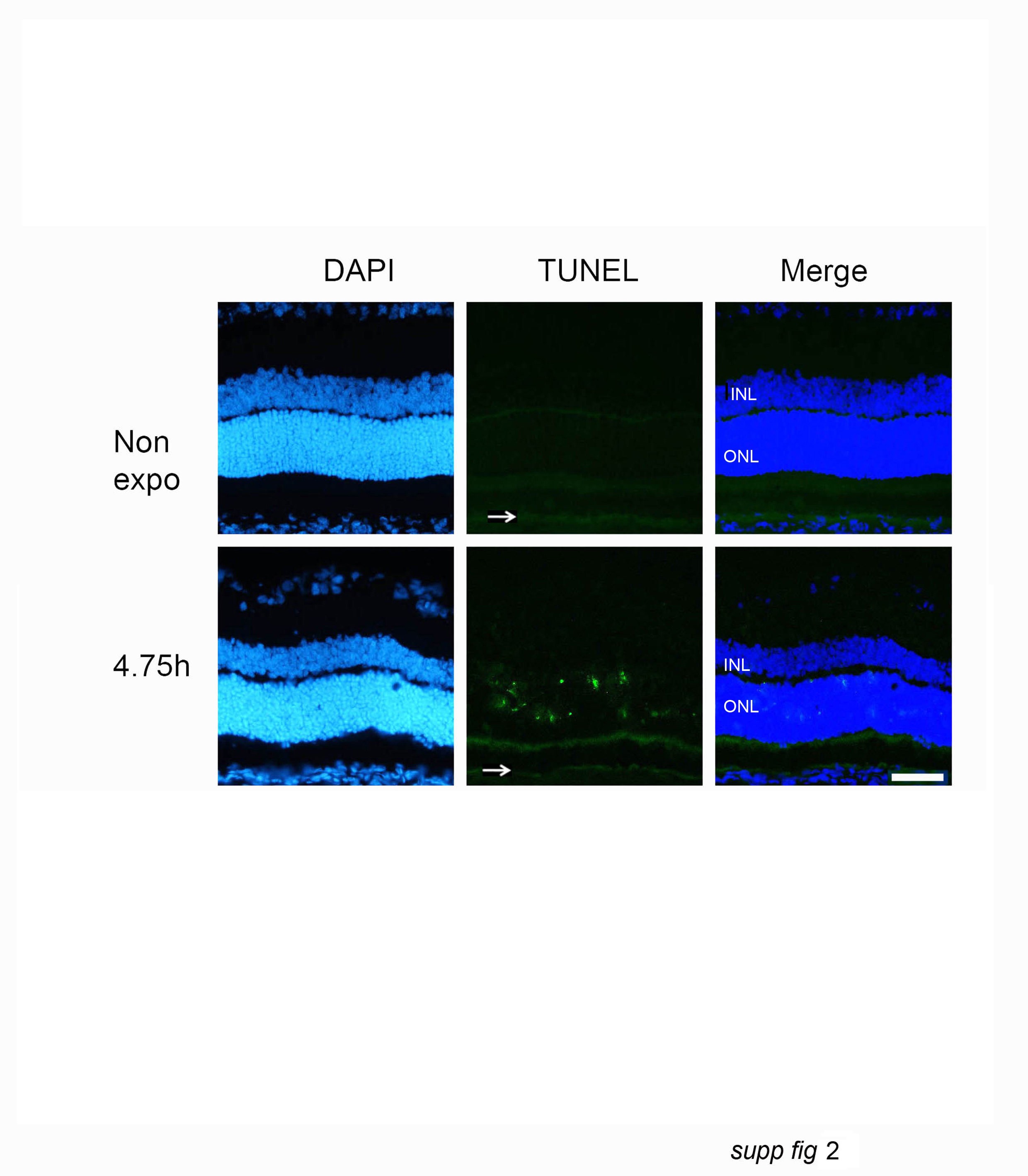

Supplement: Supplementary file 2 — Figure S2 TUNEL labelling showed no apoptosis in RPE cells. [file JCMM-21-3453-s002.jpg]

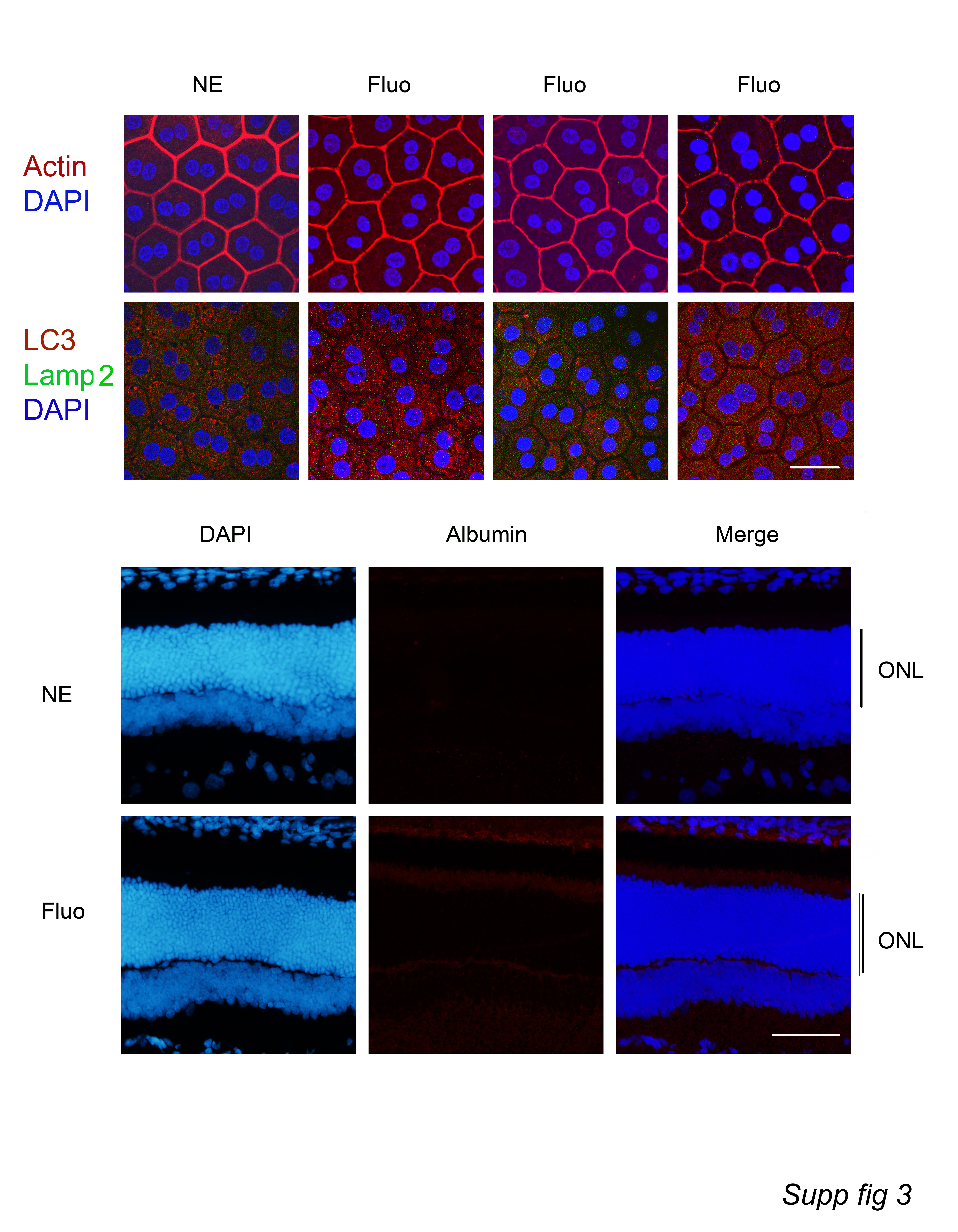

Supplement: Supplementary file 3 — Figure S3 RPE from rats exposed to 4.14 J/cm2 of fluorescent tubes. [file JCMM-21-3453-s003.jpg]
